# The impact of media-based mental health campaigns on male help-seeking: a systematic review

**DOI:** 10.1093/heapro/daae104

**Published:** 2024-09-03

**Authors:** Grant Duthie, Nicola Reavley, Judith Wright, Amy Morgan

**Affiliations:** Centre for Mental Health and Community Wellbeing, Melbourne School of Population and Global Health, The University of Melbourne, 207 Bouverie Street, Carlton, Victoria 3010, Australia; Centre for Mental Health and Community Wellbeing, Melbourne School of Population and Global Health, The University of Melbourne, 207 Bouverie Street, Carlton, Victoria 3010, Australia; Centre for Mental Health and Community Wellbeing, Melbourne School of Population and Global Health, The University of Melbourne, 207 Bouverie Street, Carlton, Victoria 3010, Australia; Centre for Mental Health and Community Wellbeing, Melbourne School of Population and Global Health, The University of Melbourne, 207 Bouverie Street, Carlton, Victoria 3010, Australia

**Keywords:** males, mass media, media, campaign, help-seeking, mental health, suicide

## Abstract

More than half of all men do not seek professional help for depression, suicide and anxiety. Although media-based campaigns represent a promising health promotion intervention to improve male help-seeking, it is unclear what communication strategies in extant mental health media-based campaigns are effective for men. The aim of this systematic review was to synthesize information about the effectiveness of these campaigns on male help-seeking outcomes. A search was conducted of electronic databases and gray literature. Studies were eligible if they examined the effectiveness of a media-based campaign targeting male help-seeking attitudes, beliefs, intentions or behaviors in relation to mental disorders, distress, suicide or self-harm. Twenty-two studies of varying quality met the eligibility criteria. Most studies targeting mental health or depression were found to positively influence male help-seeking. There were mixed results for suicide prevention campaigns. Some evidence suggests that overall, brochure-based campaigns impact help-seeking. The use of male or mixed-gender campaign imagery produced similar results. The choice of message framing appeared to influence help-seeking outcomes. Despite substantial heterogeneity in campaign approaches and difficulties isolating the effects of campaign delivery from messaging, the review indicates that media-based campaigns can play a role in improving male help-seeking for mental health difficulties. Mounting evidence suggests that messaging and delivery should align with male communication preferences. However, high-quality, targeted research is required to evaluate the circumstances in which various campaign delivery and messaging components are effective in improving male help-seeking for poor mental health and suicidality.

**Contribution to Health Promotion**

- This study found some support for media-based campaigns targeting mental health or depression in positively influencing male help-seeking. However, most studies had a high risk of bias. Brochure-based campaigns, testimonials and language tailored to male communication preferences may also improve male help-seeking.
- Evidence cautiously suggests avoiding campaigns that include specific communication approaches, such as problem-focused language and brief exposures to campaign materials using delivery modes other than brochures.
- The findings suggest the need to optimize future health promotion campaigns to male preferences to improve male help-seeking, but more research is needed.

## INTRODUCTION

An estimated 60% of adult males experiencing depression, anxiety or suicidality do not engage with mental health professionals (Australian Institute of Family Studies [AIFS], 2020). Although mental disorders are more prevalent among females (24.6%) than males (18.3%) over 12 months (Australian Bureau of Statistics [ABS], 2023b), males account for more than three-quarters of suicides in high-income countries (ABS, 2023a; Centers for Disease Control and Prevention, 2023). Similarly, males aged 16–85 years are considerably less likely to consult with a health professional about their mental health, with a recent Australian study reporting rates of 12.9% for men compared to 21.6% for similar-aged females over 12 months (ABS, 2023b). A key reason for the mental health treatment gap involves men’s reluctance to seek help (Johnson *et al*., 2012; Cleary, 2017; Oliffe *et al*., 2020), with only a quarter of men indicating they would be likely or very likely to seek help for personal or emotional concerns (AIFS, 2020). A growing body of research has indicated that traditional masculine norms, such as self-reliance, stoicism and avoiding negative emotions (e.g. sadness), may impact male help-seeking patterns (Berger *et al*., 2013; Yousaf *et al*., 2015; Seidler *et al*., 2016; Mahalik and Di Bianca, 2021). For instance, self-reliance may lead men to perceive negative emotions as a sign of weakness and hinder seeking support from friends, family members or professionals (Pirkis *et al*., 2017). Traditional masculine norms have also been associated with suicidality (Pirkis *et al*., 2017), poorer health literacy (Milner *et al*., 2019), poorer mental health outcomes (Wong *et al*., 2017) and negative attitudes toward seeking mental health support (Levant *et al*., 2009).

Numerous studies have attempted to identify effective health promotion interventions to improve male help-seeking for mental health difficulties (Sagar-Ouriaghli *et al*., 2019; Calear *et al*., 2021; Gilgoff *et al*., 2023). One such approach involves mental health media campaigns to improve male help-seeking. Media campaigns are organized activities designed to educate, convince or motivate behavior change at a population level. They use messages composed through various communication strategies (Bauman, 2000). A particular benefit afforded by media campaigns involves the publicizing of messages with a specific behavioral focus to large audiences repeatedly over a period of time in an incidental and low-cost way (Wakefield *et al*., 2010). Mental health campaigns around the world have typically aimed to reach large audiences through varied messaging in different combinations of media such as television, radio, posters, billboards, magazines, newspapers and the Internet (Pirkis *et al*., 2019; Goodwin and Behan, 2023). While mental health media campaigns exist to educate the general public about a range of mental health-related issues, at the core of many campaigns is the attempt to increase the likelihood that someone experiencing a mental health concern might reach out for help (Niederkrotenthaler *et al*., 2014; Thompson *et al*., 2020). Such campaigns often seek to influence help-seeking behaviors through proximal factors such as knowledge, intentions, beliefs, awareness, attitudes, norms, motivation and self-efficacy (Martine *et al*., 2019).

The strong potential for media campaigns to improve male help-seeking outcomes may be explained through contemporary behavior change theories such as those involving reasoned action approaches (Ajzen, 1991, 2002; Fishbein and Cappella, 2006). The theory of reasoned action suggests that a rich set of underlying beliefs involving control beliefs, normative beliefs and behavioral beliefs influence perceived behavioral control or self-efficacy, subjective norms and attitudes. These, in turn, guide the formation of a behavioral intention, subsequently motivating actual behaviors (Ajzen, 1991, 2002; Fishbein and Cappella, 2006). Reasoned action may apply within mental health media campaigns targeting male help-seeking by suggesting a causal relationship, whereby media messaging directly affects beliefs at the start of the chain. The shift in beliefs makes help-seeking appear easy to do, socially normative and to lead to good outcomes, thus influencing perceived behavioral control, subjective norms and attitudes toward seeking help. Consequently, these changes may lead to more positive help-seeking intentions that increase the likelihood that a male will access mental health treatment (Fishbein and Cappella, 2006). Media campaigns targeting other areas, such as tobacco use, have been suggested to lead to positive changes in mass populations via these theorized pathways (Wakefield *et al*., 2010).

Although traditional expressions of masculinity have been challenged and redefined by some men to incorporate more health-enhancing values (Oliffe *et al*., 2019), certain masculine ideals have been found to hinder engagement in health-promoting behaviors (Mahalik *et al*., 2007; Gordon *et al*., 2013). Accordingly, mental health marketing may be unlikely to affect help-seeking outcomes among males who hold to traditional masculine norms that lead them to suppress mental health concerns (Sharp *et al*., 2022). Several studies have highlighted the need to reframe mental health information to improve help-seeking among men (Rochlen and Hoyer, 2005; Lynch *et al*., 2018; DeBate and Gatto, 2021). Suggestions include using more humorous, non-stigmatizing language that avoids references to feelings and emotions and makes men feel valued and engaged (Robertson *et al*., 2018), incorporating solution-focused actions, promoting male qualities such as strength and reframing mental health with other terms such as ‘mental fitness’ (Lynch *et al*., 2018) and ensuring messages project a ‘sense of control’ over mental health issues (DeBate and Gatto, 2021). Despite the need for genuinely male-positive health promotion, risks emerge in attracting men with ‘male-friendly’ language that reinforces certain aspects of masculinity that result in detrimental health behaviors (Robinson and Robertson, 2010; Fleming *et al*., 2014). For instance, campaigns that ask men to ‘man up’, a colloquialism that encourages masculine ideals, may invoke positive action among males seeking to be seen as masculine but have the potential to inadvertently reinforce certain masculine traits (e.g. risk-taking) associated with adverse health consequences (Fleming *et al*., 2014). Ultimately, the nature and effectiveness of male communication strategies and preferences accommodated across extant mental health media campaigns targeting male help-seeking outcomes remain largely unexplored.

Despite the increasing interest in mental health media campaigns (Clement *et al*., 2013; Pirkis *et al*., 2019), there is a paucity of reviews that summarize the effectiveness of media campaigns targeting help-seeking, particularly among males. Some reviews have synthesized the evidence on the effectiveness of a range of interventions (including media campaigns) promoting help-seeking for mental health across various populations (Gulliver *et al*., 2012; Xu *et al*., 2018; Bu *et al*., 2020; Evans-Lacko *et al*., 2022; Johnson *et al*., 2022), including men (Seidler *et al*., 2016; Sagar-Ouriaghli *et al*., 2019). However, no robust conclusions have been made in these reviews relating to the effectiveness of mental health media campaigns. Other reviews have focused on the effect of media content on a person’s decision to seek help for mental health concerns (Goodwin and Behan, 2023) or have included help-seeking as one of several variables of interest in the context of suicide prevention or depression awareness media campaigns (Dumesnil and Verger, 2009; Torok *et al*., 2017; Pirkis *et al*., 2019). However, none of these reviews have explored gender-specific findings despite evidence for male-specific communication preferences (Robinson and Robertson, 2010). Only one paper has exclusively examined mental health campaigns for men, but this used a narrative and non-systematic approach and may not capture more recent male views about gender and masculine norms (Rochlen and Hoyer, 2005). Additionally, given the impacts of the COVID-19 pandemic on male mental health (Hadar-Shoval *et al*., 2022), exploring any recent shifts in male help-seeking responses to mental health campaigns is warranted. Collectively, these studies suggest that media-related interventions and campaigns can play a role in positively influencing mental health-related outcomes, particularly attitudes and intentions toward seeking help. Thus, further evaluation of media campaigns targeting male psychological help-seeking is needed to draw meaningful conclusions for this population.

Therefore, the current review aims to systematically search for studies evaluating media campaigns about mental health and to report on and synthesize their findings about campaign effectiveness on male help-seeking outcomes.

## METHODS

### Search strategy

This systematic review was performed according to the guidelines published by the Preferred Reporting Items for Systematic Reviews and Meta-Analysis (PRISMA) statement (Page *et al*., 2021). Studies were identified through searching three databases, PubMed, PsycINFO via OVID and Scopus, from inception to 31 December 2023, for studies providing evidence on the male help-seeking outcomes of mental health media-based campaigns. Specific search strategies were used for each database, with the search string comprising the following keywords searched by title and abstract and MeSH/Map terms: ‘help-seeking’ AND ‘mental health’ AND ‘campaign’ AND ‘media’, which included synonyms and closely related words (see Appendix A for an example of search terms used). Only studies in the English language were included in the review. A gray literature search was performed by searching Google for the top 50 hits. The websites of the top health organizations (e.g. National Health Service) and the top three charities (e.g. Beyond Blue) were also searched, and the focus was restricted to three high-income, English-speaking countries (USA, UK and Australia) for feasibility, using similar search terms adapted to fit the search tool. Additionally, efforts were made to locate additional relevant studies through hand-screening the reference lists of studies included in the review. The review protocol was registered in PROSPERO before carrying out the study (CRD42023493822).

### Eligibility criteria

Inclusion criteria were based on the PICO (Population, Intervention, Comparison, Outcome) framework. Studies were eligible if they contained either a 100% male sample or a sub-analysis comprising a male group, including comparisons of male and female help-seeking outcomes. No age restrictions were applied. However, studies of people who had already sought professional help were excluded. For this review, exposure could involve any form of an organized media-based campaign primarily targeting mental health issues to improve male help-seeking for mental health difficulties, including mental disorders (e.g. Depressive Disorders), mental distress, suicide or self-harm. Experimental interventions studied by researchers were also eligible to capture data about possible future campaigns. Campaigns that only used or studied non-media elements, such as events or in-person training, were considered ineligible.

Any methodological approach, including qualitative studies, was permitted, provided the study assessed male help-seeking outcomes. Thus, studies were excluded where no data was collected on help-seeking outcomes (e.g. purely descriptive studies with no data on male help-seeking).

Eligible outcome measures could comprise any one or combination of the following: (i) help-seeking attitudes or beliefs about treatments for mental health problems or suicide, including psychological and pharmacological treatments; (ii) help-seeking intentions, including recommending help to others; and (iii) actual help-seeking behaviors, including giving help to others, and formal or informal help-seeking from friends, family, support networks, mental health-related services or professionals and at any stage in the help-seeking engagement process. Information-seeking and help-seeking to obtain knowledge were outside the scope of this review.

### Study selection and evaluation

Studies identified through the search strategy were imported into Covidence, and duplicate studies were removed. One reviewer (G.D.) conducted title and abstract screening of all retrieved articles to identify potentially relevant studies. A second reviewer (J.W.) screened at least 20% of retrieved records. A full-text review of potentially relevant studies was conducted independently by the two reviewers (G.D. and J.W.) to determine whether the articles met the eligibility criteria. Any uncertainty about inclusion against the eligibility criteria was resolved through consensus, with a third opinion sought as required (A.M. or N.R.).

### Data extraction

After identifying included studies, data were extracted by one reviewer (G.D.) and checked by other reviewers (J.W. or A.M.), including study design, methodology, setting, study sample size, demographics, baseline characteristics, population type, campaign features, duration and mental health issues targeted, outcome measures, findings related to male help-seeking outcomes and any comments (e.g. study limitations). Any uncertainty during data extraction was resolved through consensus, with a third reviewer’s (A.M.) input as required. Results were converted into Odds Ratios or Cohen’s *d* effect sizes. Findings from qualitative, quantitative and mixed-method papers were synthesized separately. Qualitative data were summarized narratively, while quantitative data were presented narratively and tabularly.

### Study quality

Included studies utilizing a quantitative approach were critically appraised with the Effective Public Health Practice Project (EPHPP) Quality Assessment Tool for Quantitative Studies (Effective Public Health Practice Project, 1998; Thomas *et al*., 2004; Armijo-Olivo *et al*., 2012). This allowed for assessing components, including selection bias, blinding, study design, data collection methods, confounds and dropouts, with each area categorized as weak, moderate or strong. An overall quality rating was assigned to each study. Qualitative studies were assessed using the Critical Appraisal Skills Programme (CASP) Qualitative Studies Checklist (Critical Appraisal Skills Programme, 2023). Two reviewers (G.D. and J.W.) independently evaluated all eligible studies for quality, resolving discrepancies through consensus.

## RESULTS

**Fig. 1:**
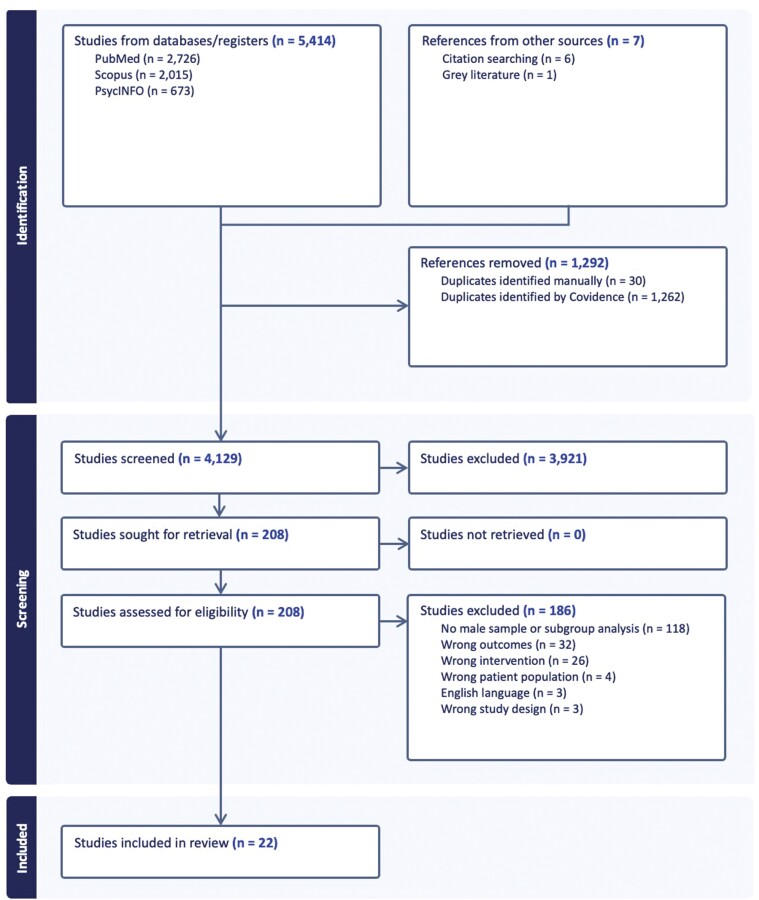
PRISMA flow chart of review process.

Figure 1 outlines the PRISMA flow diagram of the search strategy (Page *et al*., 2021). The search identified 5414 studies, of which 208 underwent full-text screening and 22 met eligibility criteria and were included in the review.

### Study characteristics

Study characteristics are presented in Table 1. Study results are available in the Supplementary Files (Supplementary Table 1). Ten studies (45.5%) examined publicly accessible media campaigns, with the remaining 12 studies (54.5%) examining a media-based intervention in an experimental setting. Publication dates ranged from 1995 to 2023, with 12 studies (54.5%) published since 2014. A variety of study designs were observed, including randomized controlled trials (RCT; 8/22; 36.4%), cohort studies, including interrupted time-series analysis (ITSA) and cross-sectional studies (11/22; 50.0%), non-randomized experimental studies (2/22; 9.1%) and qualitative (1/22; 4.5%). Most (21/22; 95.5%) were conducted in high-income countries, particularly the USA (7/22; 31.8%) and Australia (5/22; 22.7%). Only one was conducted in India (Maulik *et al*., 2019). Reported male sample sizes varied substantially, ranging from 20 to 757 402 participants, resulting in an estimated 852 027 male participants across all included studies. While the review included all age groups, a majority of studies (15/22; 68.2%) reporting age appeared to primarily sample from adult populations (aged ≥18). Despite this, there was a skew toward younger-aged adults when combining the sample ages of all studies, with a mean estimated participant age of 21.2 years.

**Table 1:** The EPHPP quality assessment tool for quantitative studies

Study	1. Selection bias	2. Design	3. Confounders	4. Blinding	5. Data collection methods	6. Withdrawals and dropouts	Global rating
Booth *et al*., 2018	S	M	S	S	S	S	S
Boucher and Campbell, 2014	W	S	W	W	S	S	L
Braun *et al*., 2023	W	S	S	S	S	S	M
Burns *et al*., 2010	W	M	W	W	S	W	L
Cheng *et al*., 2016	S	M	S	S	S	N/A	S
Daigle *et al*., 2006	S	W	W	W	M	N/A	L
Doss, 2000	W	S	W	M	S	N/A	L
Frey *et al*., 2023	W	S	S	M	S	M	M
Gilgoff *et al*., 2023	W	S	S	M	S	M	M
Hammer and Vogel, 2010	W	S	S	M	S	S	M
Hswen *et al*., 2022	W	W	M	W	M	W	L
King *et al*., 2018b	W	S	S	S	S	S	M
Maulik *et al*., 2019	M	M	M	W	S	S	M
Oliver *et al*., 2008	S	M	W	S	S	N/A	M
Rochlen *et al*., 2006	W	M	S	M	S	N/A	M
Schlichthorst *et al*., 2018	W	W	S	S	W	N/A	L
Shandley *et al*., 2010	W	M	W	W	S	W	L
Stas *et al*., 2023	W	M	M	W	S	W	L
Søgaard and Fønnebø, 1995	S	M	M	W	W	M	L
Till *et al*., 2013	S	M	W	S	S	N/A	M
Wallhed Finn *et al*., 2023	S	M	W	S	S	N/A	M

Component Ratings: 1A. Are the individuals selected to participate in the study likely to be representative of the target population? 1B. What percentage of selected individuals agreed to participate? 2A. Study design; 2B. Was the study described as randomised? 2C. Was the method of randomisation described? 2D. Was the randomisation process appropriate? 3A. Were there important differences between groups prior to the intervention? 3B. What percentage of relevant confounders were controlled? 4A. Were the outcome assessors aware of the intervention status of participants? 4B Were the participants aware of the research question? 5A. Were data collection tools shown to be valid? 5B. Were data collection tools shown to be reliable? 6A. Were withdrawals and drop-outs reported in terms of numbers/reasons? 6B. Indicate the percentage of participants completing the study. Global Rating Strong (S) = no weak (W) ratings; Moderate (M) = one weak rating; Low (L) = two or more weak ratings; N/A = not applicable.

### Campaign evaluation

Seven studies assessed help-seeking attitudes, 12 assessed intentions to seek help and 8 assessed actual help-seeking behaviors. Among all included studies, four (18.2%) assessed more than one type of outcome. There was substantial heterogeneity in the way campaigns evaluated help-seeking outcomes. While three studies (13.6%) used the General Help-Seeking Questionnaire (GHSQ) to measure help-seeking intentions, and three studies (13.6%) used the Attitudes Towards Seeking Professional Psychological Help Scale (ATSPPHS) to measure help-seeking attitudes, the remaining 16 studies (72.7%) used various other measurement tools including self-created scales.

### Study quality

As shown in Tables 1 and 2, 2 studies (9.1%) included in this review were rated high quality, with 11 studies (50.0%) rated moderate quality and 9 studies (40.9%) rated low quality, indicating that most studies are at risk of selection bias.

**Table 2:** The CASP quality assessment tool for qualitative studies

Study	CASP criterion										
	1	2	3	4	5	6	7	8	9	10	Total Score
King *et al*., 2018a	2	2	2	2	2	0	2	2	2	2	18

CASP criterion: 1. Was there a clear statement of the aims of the research?; 2. Is a qualitative methodology appropriate?; 3. Was the research design appropriate to address the aims of the research?; 4. Was the recruitment strategy appropriate to the aims of the research?; 5. Was the data collected in a way that addressed the research issue?; 6. Has the relationship between researcher and participants been adequately considered?; 7. Have ethical issues been taken into consideration?; 8. Was the data analysis sufficiently rigorous?; 9. Is there a clear statement of findings?; 10. How valuable is the research?; CASP critical score as cited in Njau *et al.* (Njau *et al*., 2019): the criterion is met = 2; the criterion is partially met = 1; the criterion not applicable, not met or not reported = 0; Total score 20 = high quality; 16–19 moderate quality; ≤15 low quality.

### Campaign focus

As shown in Table 3, most campaigns focused on various mental health concerns (6/22; 27.3%) or mental health combined with suicide prevention (5/22; 22.7%). Among them, six studies found an improvement in male help-seeking outcomes (i.e. attitudes, intentions and behaviors), whereas five studies did not find an effect. Concerning depression-specific (4/22; 18.2%) or depression combined with suicide prevention campaigns (2/22; 9.1%), mixed results were also evident. Specifically, four studies revealed an effect, and two others showed no effect on male help-seeking outcomes. Another four studies (18.2%) examined suicide prevention-specific campaigns. Of them, three studies found no effect on male help-seeking, whereas one study did report an improvement (Oliver *et al*., 2008). No effect was found for the single study (4.5%) targeting alcohol use disorder (Wallhed Finn *et al*., 2023).

**Table 3:** Campaign features of studies included in the review

Author (Date)	Mental health problem focus	Target group	Mode of delivery	Intensity	Intervention visuals	Intervention messaging
Booth *et al*. (2018)	Mental health (non-specific)	General public	Online (social media)	Long	Campaign hosted by a prominent Canadian female Olympic athlete	Mixed-gender language (e.g. donation offered for each interaction with campaign username ‘Bell Let’s Talk’, female athlete recounts her struggle with depression and encouraged the public to start a conversation to break the silence around mental health issues and to increase mental health awareness across Canada)
Boucher & Campbell (2014)	Depression	College students	Billboard viewed online	Brief	NR	Problem-focused, mixed-gender language (e.g. ‘Depression is a brain disease’)
Braun *et al*. (2023)	Suicide	Adolescents	Video	Brief	Featured a 17-year-old boy	Male-targeted, gain-framed language (e.g. personal story about a past suicidal crisis and his way of overcoming it, highlighting how he came through it, detailing the importance of proactive help-seeking)
Burns *et al*. (2010)	Mental health (non-specific)	Australian young people but the campaign aims to appeal to young men	Online (website)	Long	Featured diverse male and female characters with different personalities (e.g. ‘the cool guy’, ‘the loose cannon’ or the ‘straight-up guy’)	Gain and loss-framed language (e.g. examines consequences of choices made during the game to manage mental health difficulties)
Cheng *et al*. (2016)	Mental illness (non-specific)	General public	Radio, websites, newspapers, posters in bus shelters, subways and public transport stations	Long	Featured imagery of males and females	Mixed gender, gain-framed language (e.g. personal stories about how a psychiatric hospital transformed lives)
Daigle *et al*. (2006)	Suicide	Males	Newspaper articles, radio, television advertisements	Long	NR	Problem-focused, male-targeted language (e.g. ‘Pain is not gender-specific—yet 80% of suicides are committed by men’, aimed at changing the belief that males should not feel pain and are unable to express their suffering)
Doss (2000)	Mental illness (non-specific)	Males	Advertising brochure	Brief	No imagery	Gain-framed, male-sensitive language (e.g. ‘We are not about feelings, we are about actions’)
Frey *et al*. (2023)	Depression and suicide	Males	Online (website)	Long	Featured a fictitious male host, Dr Rich Mahogany	Gain-framed, male-sensitive language (e.g. male-oriented language and metaphor, as well as action-oriented styles of treatment such as ‘Gentlemental Health®101—Sleep—When Catching Z’s is Harder Than Catching a 20 lb. Trout’, ‘20 Point Head Inspection’ a play on words referring to a mechanic checking under a car’s hood, and sports-themed references to reframe therapy such as ‘It’s like a driving range for your mental game’)
Gilgoff *et al*. (2023)	Depression and suicide	Males	Online (website)	Long	Featured a fictitious male host, Dr Rich Mahogany	Gain-framed, male-sensitive language (e.g. male-oriented language and metaphor, as well as action-oriented styles of treatment such as ‘Gentlemental Health®101—Sleep—When Catching Z’s is Harder Than Catching a 20 lb. Trout’, ‘20 Point Head Inspection’ a play on words referring to a mechanic checking under a car’s hood, and sports-themed references to reframe therapy such as ‘It’s like a driving range for your mental game’)
Hammer and Vogel (2010)	Depression	Males	Brochure	Brief	Featured masculine-looking men	Gain-framed, male-sensitive language (e.g. ‘masculine depression’, describing counseling as a cost-effective, solution-focused and client-directed team effort)
Hswen *et al*. (2022)	Depression	General public	Online search engine advertising	Brief	No imagery	Problem-focused, male-targeted language (e.g. ‘Are you sad? Men are poorly followed for depression. Follow this link to find out more about yourself’)
King *et al*. (2018a)	Mental health (non-specific) and suicide	Males	Video (documentary)	Long	Documentary presented by male radio and television personality and featured diverse males modeling positive health behaviors, such as seeking help or sharing personal problems with others.	Gain and loss-framed language (e.g. examined link between masculinity and mental health and provided examples about how seeking help improved a person’s life for the better)
King *et al*. (2018b)	Mental health (non-specific) and suicide	Males	Video (documentary)	Long	Documentary presented by male radio and television personality and featured diverse males modeling positive health behaviors, such as seeking help or sharing personal problems with others.	Gain and loss-framed language (e.g. examined link between masculinity and mental health and provided examples about how seeking help improved a person’s life for the better)
Maulik *et al*. (2019)	Depression, anxiety, stress and suicide	General public	Informational material and videos shown at door-to-door visits; posters made available at public places such as administrative buildings, schools and primary health services; theater production staged across all villages	Long	Featured mixed-gender imagery	NR
Oliver *et al*. (2008)	Suicide	General public	Billboards, posters on buses and shops, radio advertisement	Long	Featured indeterminate gender imagery	Mixed gender, gain-framed language (e.g. ‘Suicide is preventable. Its causes are treatable. For immediate help call (emergency number)’)
Rochlen *et al*. (2006)	Depression	Males	Brochure	Brief	Featured diverse males	Gain-framed, male-sensitive language (e.g. ‘It takes courage to ask for help’)
Schlichthorst *et al*. (2018)	Mental health (non-specific) and suicide	Males	Video (documentary), social media, newsprint, radio and website	Long	Documentary presented by male radio and television personality and featured diverse males modeling positive health behaviors, such as seeking help or sharing personal problems with others.	Gain and loss-framed language (e.g. examined link between masculinity and mental health and provided examples about how seeking help improved a person’s life for the better)
Shandley *et al*. (2010)	Mental health (non-specific)	Australian young people but the campaign aims to appeal to young men	Online (website)	Long	Featured diverse male and female characters with different personalities (e.g. ‘the cool guy’, ‘the loose cannon’ or the ‘straight-up guy’)	Gain and loss-framed language (e.g. examines consequences of choices made during the game to manage mental health difficulties)
Stas *et al*. (2023)	Mental health (non-specific) and suicide	Males	Online (website), videos	Long	Featured male imagery	Problem-focused, male-targeted language (e.g. ‘Get out of your head’ and featured stories of men struggling with mental health)
Søgaard and Fønnebø (1995)	Mental illness (non-specific)	General public	Video (television show)	Long	NR	NR
Till *et al*. (2013)	Suicide	General public but focused on men	Billboards, posters and placards sent to locations such as community centers and psychosocial institutes, info screens in railways and traffic junctions	Long	Imagery of positive everyday life experiences (e.g. a child’s drawing, a restaurant menu, a postcard from a friend)	Male-targeted, gain-framed language (e.g. ‘There are a lot of reasons to love life. If you are currently unable to find a reason: Call (hotline) and let’s talk about it!’)
Wallhed Finn *et al*. (2023)	Alcohol Use Disorder	Males	Television and Internet advertisements, local activities, posters, pamphlets on social media and in public places, short movies	Long	NR	Male-targeted, gain-framed language (e.g. positive consequences of seeking treatment for alcohol use such as gaining respect from others)

NR = not reported; Long = longer duration of campaign material exposure (i.e. typically >1 hour) or where there is a possibility for repeated exposure to campaign materials; Brief = short duration of campaign material exposure (i.e. typically <1 hour); Nixed-gender imagery = featuring images of men and women; Gain-framed = focusing on the positive consequences behavior; Loss-framed = focusing on the negative consequences of behavior; Problem-focused = helping men to identify with mental health difficulties; Male-sensitive = incorporates male communication preferences (e.g. humor, solution-focused, avoidance of emotions); Male-targeted = language that focuses on men but does not accommodate their communication preferences; Mixed-gender language = language that is not tailored specifically to men or women.

### Campaign mode of delivery

Among the primary media channels used in studies were multiple modes (8/22; 36.4%), online modes including Internet advertisements and websites (5/22; 22.7%), video (4/22; 18.2%), print brochures (3/22; 13.6%), billboards (1/22; 4.5%) and social media (1/22; 4.5%). There were mixed results for website-based campaigns on male help-seeking outcomes, with low-quality studies reporting either no within-group effect for an Internet-based game (Burns *et al*., 2010; Shandley *et al*., 2010) or no between-group effect for an Internet-based advertisement (Hswen *et al*., 2022). However, two moderate-quality RCTs of the Man Therapy website found that males viewing either the experimental or control website comprising anonymous screening experienced improvements in their help-seeking outcomes at 12 weeks follow-up (Frey *et al*., 2023). The other study found that controlling for several covariates, those who viewed the experimental website had greater actual help-seeking behaviors than those in the control condition at 2 weeks’ follow-up (Gilgoff *et al*., 2023). However, such findings should be interpreted cautiously as the study may have broken group randomization due to its analytic approach. Relatedly, a strong-quality ITSA of a social media campaign found an increase in new adolescent and young adult male mental health visits in the post-campaign period (Booth *et al*., 2018).

Mixed findings were also apparent in campaigns using videos. King *et al*. conducted two moderate-quality studies of the Man Up documentary (King *et al.*, 2018a,b). They found significant improvements at 4 weeks’ follow-up in help-seeking intentions among males viewing a mental health documentary relative to controls (King *et al*., 2018b). Most male documentary viewers reported qualitative improvements in help-seeking and offering behaviors at follow-up (King *et al*., 2018a). Conversely, two moderate to low-quality studies found no improvement in male help-seeking at 1-month follow-up measures (Søgaard and Fønnebø, 1995; Braun *et al*., 2023).

More consistently favorable findings were shown in brochure studies (Doss, 2000; Rochlen *et al*., 2006; Hammer and Vogel, 2010). Three moderate to low-quality RCTs, showed that males reading brochures had improved post-test attitudes toward seeking professional help (Rochlen *et al*., 2006; Hammer and Vogel, 2010) or higher amenability to try psychological services at immediate post-test relative to controls (Doss, 2000). In contrast, one billboard study found no effect on help-seeking intentions at post-test (Boucher and Campbell, 2014).

Evaluation of multi-mode campaigns also reported mixed impacts on male help-seeking outcomes. Three strong to moderate-quality studies reported improved male help-seeking outcomes. These comprised campaigns delivered via a combination of advertisements on the radio, websites, newspapers and posters (Cheng *et al*., 2016) or informational materials, posters, videos and theater shows (Maulik *et al*., 2019), or billboards, posters and radio advertisements (Oliver *et al*., 2008). In contrast, two moderate-quality studies of multi-mode campaigns found no improvement in male help-seeking behaviors using a mix of billboards, posters, placards and info screens (Till *et al*., 2013) or advertisements on television, Internet, posters, pamphlets and videos (Wallhed Finn *et al*., 2023). Similarly, three low-quality studies found no effect on help-seeking outcomes. These studies used various combinations of media channels, including newspaper articles, radio and television advertisements (Daigle *et al*., 2006), websites and videos (Stas *et al*., 2023) or a documentary, social media, website, radio and newsprint (Schlichthorst *et al*., 2018).

### Campaign intensity

There were mixed findings regarding the intensity of campaign exposure on male help-seeking outcomes. Two categories were assigned. Short exposure involved exposure to the campaign material, typically lasting less than an hour. In contrast, long exposure involved prolonged exposure to campaign materials, typically lasting more than an hour or where there was a possibility of repeated exposure to the campaign. Three moderate to low-quality studies showed that short-intensity campaigns were not effective on male help-seeking. Specifically, one study revealed no within-group effect among males having a single exposure to a brief suicide prevention video (Braun *et al*., 2023), and two other studies showed no between-group effect for brief messages at post-test (Boucher and Campbell, 2014; Hswen *et al*., 2022). Help-seeking increases were found for seemingly brief brochures in one low-quality study (Doss, 2000) and two moderate-quality studies (Rochlen *et al*., 2006; Hammer and Vogel, 2010). Concerning other experimental campaigns using longer-intensity media, King *et al.* found improvements in male help-seeking across two moderate-quality studies of the 3-hour-long documentary Man Up (King *et al*., 2018a,b). Similarly, two moderate-quality studies of the Man Therapy website, where participants could view the website multiple times, found an improvement (Frey *et al*., 2023; Gilgoff *et al*., 2023). Another long exposure and low-quality experimental online media campaign found no effect (Stas *et al*., 2023). Neither did the low-quality studies of Reach Out Central, which included long exposure through repeated gameplay messaging throughout the day (Burns *et al*., 2010; Shandley *et al*., 2010). Conversely, among publicly accessible campaigns, strong to low-quality studies, where there is a possibility for repeated viewing and prolonged exposure, five found no effect on male help-seeking (Søgaard and Fønnebø, 1995; Daigle *et al*., 2006; Till *et al*., 2013; Schlichthorst *et al*., 2018; Wallhed Finn *et al*., 2023). In contrast, another four found an improvement in male help-seeking (Oliver *et al*., 2008; Cheng *et al*., 2016; Booth *et al*., 2018; Maulik *et al*., 2019).

### Campaign imagery

A majority (16/22; 72.7%) of studies reported on their use of visual elements in the media-based campaign, where seven used diverse males in their campaign imagery (31.8%). Among studies mainly using male imagery, three moderate to low-quality studies found no within-group effect on help-seeking when viewing videos of males discussing various mental health topics (Schlichthorst *et al*., 2018; Braun *et al*., 2023; Stas *et al*., 2023). At the same time, four moderate-quality studies featured videos and imagery of diverse males, including typically masculine males, mental health professionals, men’s health experts, hosts including media personalities or fictitious characters and role models for beneficial health behaviors, such as sharing personal problems with others or seeking help. The results showed that male viewers improved their help-seeking outcomes at follow-up measures (King *et al*., 2018a,b; Frey *et al*., 2023; Gilgoff *et al*., 2023). Similarly, two moderate-quality studies compared campaign media featuring either imagery of males alone or with mixed-gender imagery (9.1%). At post-test measures, Hammer and Vogel found that males viewing masculine-looking men in testimonial portraits when reading a male-specific brochure experienced greater reductions in self-stigma toward seeking help but not help-seeking attitudes when compared to males viewing testimonial portraits of both males and females in a gender-non-specific brochure (Hammer and Vogel, 2010). Contrastingly, Rochlen *et al*. found no between-group effects in the help-seeking outcomes of males viewing different brochures containing either male-specific imagery or male and female actual or drawn imagery; instead, all conditions led to significant improvements in post-test measures (Rochlen *et al*., 2006).

Five studies (22.7%) used mixed-gender imagery (i.e. featuring images of men and women), with mixed findings. Among them, three strong to moderate-quality studies found improved male help-seeking outcomes from campaigns that reported using imagery of people sharing their personal stories about mental health not specific to either gender (Cheng *et al*., 2016; Maulik *et al*., 2019) or reported using indeterminate gender imagery (Oliver *et al*., 2008). Despite this, low-quality studies examining the Reach Out Central game, featuring diverse male and female characters, did not find a within-group effect on young male help-seeking outcomes (Burns *et al*., 2010; Shandley *et al*., 2010). In the remaining studies, one study (4.5%) reported using a prominent female Olympic athlete as a campaign host, revealing a significant increase in adolescent and young adult male help-seeking behaviors (Booth *et al*., 2018). Only one study (4.5%) featured no images of people but incorporated imagery depicting positive everyday life experiences. However, it did not show a within-group effect on help-seeking calls among males (Till *et al*., 2013). The remaining six studies (27.3%) did not use any imagery or did not report imagery used.

### Campaign messaging

Substantial heterogeneity of messaging styles was found across different campaigns, with several categories assigned, as shown in Table 4. Five studies (22.7%) primarily used gain-framed combined with male-sensitive language, whereas two studies (9.1%) included gain-framed language but did not target men specifically. Problem-focused framing with male-targeted language was used by three studies (13.6%). Three studies (13.6%) used a combination of gain and loss-framed language and targeted men, while two studies (9.1%) used gain combined with loss-framed without a specific male focus. Similarly, another three studies (13.6%) used gain-framed language while targeting men. One study (4.5%) used problem-focused with mixed-gender language but did not find a between-group effect (Boucher and Campbell, 2014). Another study (4.5%) used mixed-gender language and found a within-group effect on young males (Booth *et al*., 2018). The remaining studies could not be categorized based on messaging (2/22; 9.1%).

**Table 4:** Campaign messaging category definitions

Messaging style	Definition	Example
Gain-framed messaging	Language that focuses on the positive consequences of behavior	Campaign messaging: ‘There are a lot of reasons to love life’ (Till *et al*., 2013)
Loss-framed messaging	Language that focuses on the negative consequences of behavior	Campaign description of a Reach Out Central gameplay scenario: ‘If mood is low, making friends is more difficult’ (Shandley *et al*., 2010)
Problem-focused language	Language that helps men to identify with mental health difficulties	Campaign messaging: ‘Are you sad?’ (Hswen *et al*., 2022)
Male-sensitive language	Language that incorporates male communication preferences (e.g. humor, solution-focused, avoidance of emotions)	Campaign messaging: ‘We are not about feelings, we are about actions’ (Doss, 2000)
Male-targeted language	Language that focuses on men but does not accommodate their communication preferences	Campaign messaging: ‘80% of suicides are committed by men’ (Daigle *et al*., 2006)
Mixed-gender language	Language that is not tailored specifically to men or women	Campaign description: ‘The campaign encourages the public to start a dialogue to break the silence around mental illness and to support mental health awareness across Canada’ (Booth *et al*., 2018)

Mixed results were shown among moderate to low-quality studies with gain-framed and male-sensitive language on male help-seeking outcomes (Doss, 2000; Rochlen *et al*., 2006; Hammer and Vogel, 2010; Frey *et al*., 2023; Gilgoff *et al*., 2023). Doss found that a gender-sensitive brochure describing therapy in various ways, such as that it will not initially focus on emotions, was more effective than a non-gender-sensitive brochure and control condition in producing help-seeking outcomes at post-test measures (Doss, 2000). Nonetheless, Frey *et al*. found no between-group effect for males viewing the Man Therapy website that contained various approaches, such as framing help-seeking as a strength, than those only viewing an anonymous online screening and referral website (Frey *et al*., 2023). However, at a 2-week follow-up, a secondary analysis by Gilgoff *et al*. (Gilgoff *et al*., 2023) showed that when analyses adjusted for several covariates, males who viewed Man Therapy reported significantly more instances of actual professional help-seeking behavior but not non-professional help-seeking behaviors compared to the control website. Conversely, Rochlen *et al*. found no interaction effect of time by group for men receiving a brochure using male-specific information about depression, male testimonials about depression and male-sensitive language compared to those receiving non-gender-specific brochures (Rochlen *et al*., 2006). However, when also comparing a gender-specific and non-specific brochure, Hammer and Vogel showed that there was a significant interaction of time by group for help-seeking attitudes in favor of a brochure with male testimonials and male-sensitive language such as language consistent with traditional masculine gender roles (e.g. mental health consultant) and a medical-model explanation for depression (Hammer and Vogel, 2010).

Significant increases in male help-seeking behaviors were shown in both studies that appeared to use a mixed-gender focus with gain-framed language, including a campaign that used personal stories about how a psychiatric hospital transformed lives (Cheng *et al*., 2016) and another campaign featuring the message ‘Suicide is preventable. Its causes are treatable. For immediate help call (emergency number)’ (Oliver *et al*., 2008). Whereas all campaigns that used problem-focused approaches with male-targeted language (e.g. encouraging men to identify pain, asking men if they are sad or stories about men with mental health struggles) found no effect on male help-seeking (Daigle *et al*., 2006; Hswen *et al*., 2022; Stas *et al*., 2023). Similarly, none of the moderate-quality studies using male-targeted approaches with gain-framed language (e.g. the benefit of gaining respect from treatment seeking, an adolescent sharing about mastering a past suicidal crisis or indicating that there are many reasons to love life) found a within-group effect on male help-seeking (Till *et al*., 2013; Braun *et al*., 2023; Wallhed Finn *et al*., 2023).

Mixed results were apparent in studies containing both gain and loss-framed messaging together. Three of the five did not find a within-group effect on male help-seeking, including the studies of the single-player Internet game Reach Out Central that sought to teach participants life skills, strategies to improve mood and the consequences of choices made to manage mental health difficulties across real-life scenarios (Burns *et al*., 2010; Shandley *et al*., 2010). Despite this, only the two moderate-quality studies by King *et al*. (King *et al*., 2018a,b) found significantly higher help-seeking outcomes at follow-up measures among males who watched the documentary relative to controls. In contrast, the study by Schlichthorst *et al*. (Schlichthorst *et al*., 2018) of the same documentary found no within-group effect on male help-seeking at post-test measures, which may be attributable to the higher help-seeking baseline scores among viewers of the Man Up documentary.

## DISCUSSION

Given the growing research interest in the role of media-based campaigns for public health interventions among males, this review aimed to synthesize the findings about the effectiveness of media-based mental health campaigns on male help-seeking outcomes. To the authors’ knowledge, this review is the first to evaluate male help-seeking in the context of media-based campaigns. While there are promising indications for campaigns using male communication strategies to promote help-seeking, some caution is required in interpreting the results. Included studies tended to be rated low quality, and several did not show positive results. However, methodological limitations may hinder most studies showing null effects, such as small sample sizes, large attrition rates or large differences in group sample sizes. Despite this, the evidence collectively suggests that campaigns targeting mental health or depression can positively influence male help-seeking, including having an immediate effect on actual help-seeking behaviors. Overall, some supportive evidence was found for specific campaign components, including brief exposures to brochure-based campaigns, male or mixed-gender campaign imagery, testimonials and gain-framed messages combined with male-sensitive language as part of the campaign messaging. The results cautiously suggested avoiding other components, including problem-focused language, gain-framed messages targeted at men that do not accommodate their language preferences and brief campaign exposures using delivery modes other than brochures.

Despite the mental health impacts of the COVID-19 pandemic, campaigns run during the 2020–22 years did not affect male help-seeking (Hswen *et al*., 2022; Braun *et al*., 2023; Stas *et al*., 2023). However, it is worth noting that the causes of male help-seeking appear to have shifted during the pandemic, with studies suggesting more help-seeking related to high anxiety than suicide attempts (Moore *et al*., 2022). Interestingly, none of the campaigns run during the pandemic focused on anxiety conditions, which may explain the absence of help-seeking improvements. Ultimately, more research is needed to ascertain any shifts in male help-seeking reactions to campaigns following the pandemic.

At a campaign focus level, the results indicate some evidence for improvements in male help-seeking among media-based campaigns addressing mental health and depression with or without suicide prevention. Although there were some exceptions, this may be due to a lack of power in several instances. In contrast, little evidence exists for media-based campaigns targeting suicide prevention on their own in improving help-seeking outcomes. These findings appear discrepant from other reviews that included male and female populations (Torok *et al*., 2017; Pirkis *et al*., 2019). However, studies that did not find an improvement may have also been underpowered. Further research with larger sample sizes is needed to confirm these findings.

When examining the mode of campaign delivery, some evidence was found that multi-channel campaigns can improve help-seeking, but this may not always be the case. Nonetheless, according to Dumesnil and Verger (Dumesnil and Verger, 2009), the impact of multi-channel campaigns may relate to their ability to increase message precision, while their exposure duration may also allow for increased message reinforcement. Despite this, not all included studies were effective when they included extended participant exposure to the campaign materials. Notably, most campaigns that did not show an effect of prolonged exposure duration measured either help-seeking intentions or actual help-seeking behaviors rather than attitudes. This aligns with reasoned action theories, positing that modifying attitudes toward help-seeking via media-based campaigns may be easier than influencing intentions or actual behaviors (Ajzen, 1991, 2002; Fishbein and Cappella, 2006).

There was limited evidence that website and video-based campaigns were effective despite being the most common delivery modes. These findings appear discrepant from other review findings that included male and female populations (Goodwin and Behan, 2023) or non-media-based interventions (Sagar-Ouriaghli *et al*., 2019). As a more traditional mode of health communication (Jansen *et al*., 2021), the available evidence suggests that brief brochure-based campaigns can improve help-seeking outcomes, consistent with other review findings (Sagar-Ouriaghli *et al*., 2019). However, some studies may be hindered by poor ecological validity, relying on student samples. Despite this, brochures were key components of all effective brief exposure campaigns, with no evidence suggesting other delivery modes were effective for brief exposures. These results suggest that males may not require repeated or prolonged campaign exposure when the content is more informational, allowing them to process brief materials at their own pace. Informational materials may also be less personal or intrusive and enable males to understand their difficulties in context and discern the possible consequences (Sagar-Ouriaghli *et al*., 2019). This makes brochures seemingly more conducive to marketing mental health help-seeking to males than other modes.

Among campaigns using imagery, the results indicate that male help-seeking outcomes may be equally influenced by male or mixed-gender imagery. This appears inconsistent with the research suggesting that imagery with females may confuse males reading health-related messages (Ryan *et al*., 2019). In contrast, positive depictions of males (e.g. in leadership positions) may appeal more to males in health promotion initiatives (Ryan *et al*., 2019). Given the human tendency to seek schema-congruent information (Meyers-Levy and Tybout, 1989), males adhering to traditional masculine norms may prefer a more masculine presentation of help-seeking that aligns with existing norms about male gender roles (e.g. strength and taking control) to ensure messaging does not threaten their masculine identity (Lynch *et al*., 2018). However, research is needed to isolate the effects of imagery relative to other campaign elements, as it may be conflated with campaign delivery.

In terms of campaign messaging, combined gain and loss-framed messages appeared to have limited impacts on help-seeking outcomes. In contrast, no evidence was found for the benefits of using problem-focused language. Given the importance of masculine values such as emotional control (Mahalik and Di Bianca, 2021), it is possible that campaigns using problem-focused language may evoke reactance due to a fear of aversive consequences (e.g. having a mental illness) or a perception of the message being a direct challenge to masculine norms. Interestingly, male-targeted and gain-framed messaging were shown to have null effects. This concurs with research showing that some positive messages aimed at increasing help-seeking can have the reverse effect by potentially activating stigmatizing beliefs about help-seeking (Lienemann *et al*., 2013).

Campaigns that included testimonials or narratives about topics relating to mental health or suicide showed a positive effect on help-seeking in general. This is consistent with other research showing that narrative communication may facilitate better health outcomes due to their ability to exert more persuasive effects (Chang, 2008; Moyer-Gusé and Nabi, 2010; Murphy *et al*., 2013). Narratives may be perceived as more subtly influential and less condescending than explicit health advocacy efforts with non-narrative approaches (Oschatz *et al*., 2022), which use evidence-based reasoning and logic to argue a claim (Kreuter *et al*., 2007). Despite this, two brochure-based campaigns did not find superior effects of brochures containing testimonials compared to non-narrative information. This suggests that narratives may be beneficial but are not necessary to affect male help-seeking outcomes when using brochures. Future research could confirm these findings.

In contrast, campaign effectiveness appears to increase when a combination of gain-framed approaches and male-sensitive language is implemented, with all such campaign messages showing an effect. The effectiveness of gain-framed messaging combined with male-sensitive language aligns with recommendations suggesting that positive messaging may assist in destigmatizing help-seeking among males (Lynch *et al*., 2018). Effective male-sensitive messages appeared to incorporate various language components, including humor, framing help-seeking as strength, appeals to masculine values (e.g. courage), avoiding mentions of feelings and emotions, giving a perceived sense of control over difficulties, non-stigmatizing language (e.g. ‘masculine depression’) and appeals to logic. These promising results on male-sensitive language appear consistent with the broader literature, which recognizes the need to reframe mental health language to gain traction among men (Rochlen and Hoyer, 2005; Lynch *et al*., 2018; DeBate and Gatto, 2021). The success of male-sensitive language may occur through its ability to balance traditional masculinities by including masculine values conducive to mental health (e.g. seeking help is a strength). At the same time, it can also destigmatize mental health by avoiding negative connotations associated with seeking help (e.g. focusing on emotions; Sharp *et al*., 2022). Despite these potential benefits of male-sensitive language, studies comparing message types did not demonstrate superior effects of male-sensitive relative to mixed-gender messaging and difficulties remain in isolating the specific influence of messaging. Relatedly, campaigns that used mixed-gender language alone or combined with gain-framed messages were shown as effective. Taken together, these results indicate that tailoring messages to males may be beneficial but not necessary to promote male help-seeking. Still, more research is needed to tease out the effects of gain versus loss-framing in the context of help-seeking messaging.

Interestingly, most studies that examined actual help-seeking behaviors found an effect. This indicates that campaigns can affect immediate behavioral changes under the right conditions, even with brief campaign exposures. In the few studies examining multiple outcomes, results were also consistent with the theorized pathways for affecting male help-seeking, with similar results shown across attitudes, intentions or actual behaviors. Despite the role of traditional masculinities in help-seeking outcomes (Seidler *et al*., 2016), it does not appear that any effective campaigns directly challenged masculine norms to influence help-seeking. If the theorized pathways were in effect, it is possible that effective campaigns were able to alter men’s attitudes toward help-seeking by reframing the behaviors to be consistent with traditional masculinities. Alternatively, marketing services that accommodate traditional masculine norms and do not require men to alter their adherence to such norms (Smith *et al*., 2008). Interpreted this way, reasoned action approaches (Ajzen, 1991, 2002; Fishbein and Cappella, 2006) appear to be a promising model for understanding male help-seeking. They suggest that attitude changes may motivate changes along the causal chain and occur without altering adherence to traditional masculinities.

## LIMITATIONS

Ultimately, this review’s findings should be interpreted with caution given the heterogeneity of included studies, which restricted any discussion of findings to broad generalizations. Additionally, several studies had design limitations and confounding variables, such as the presence of non-media components, and it may not be possible to isolate the effects of campaign messaging from the delivery. While there was some variation in sample characteristics across studies, studies were primarily sourced from high-income countries and relied on convenience sampling. Given that males are not a homogenous group, the results may not be generalizable to other men. There were also diverse approaches to evaluating campaign effectiveness, including commonly used measures (e.g. GHSQ) and self-created scales, which increase the likelihood of measurement error across studies. Furthermore, only some campaigns conducted follow-up measurements beyond three months, which makes it difficult to determine the long-term effects of campaigns on help-seeking outcomes.

Although a reasonable number of studies were identified in the search strategy, the largest study exclusion reason was the absence of male subgroup analysis, revealing a possible selection bias that may hinder this review’s external validity. Other relevant studies may have been excluded because of this review’s approach (e.g. articles not written in English or the limited screening of retrieved studies by the second reviewer). Also, most included studies could not be considered methodologically robust, with only two included studies assigned a strong-quality rating, which weakens the internal validity of this review’s results. It is also worth acknowledging the potential for publication bias (i.e. where studies with null effects are less likely to be published) and issues around a lack of publishing of the results of historical mental-health campaigns, which may impact the validity of this review’s findings.

## FUTURE DIRECTIONS

Despite some promising indications for the role of media-based mental health campaigns in improving male help-seeking outcomes, the evidence is still equivocal. Thus, there is a need for more high-quality research that carefully evaluates mental health media-based campaigns and the circumstances in which various campaign components are effective for male help-seeking. Future research may also benefit from more theoretically informed approaches to campaign development and rigorous campaign testing before launch to ascertain its effectiveness. This will assist in growing the evidence base for functional campaign components and ensure more cost-effective and targeted campaign development amid greater funding scarcity. Work is also needed to understand the effects of campaigns targeting other specific mental disorders while ascertaining the robustness of the current review’s findings.

Drilling down further to determine the optimal campaign components that influence male help-seeking is necessary. Evaluations should also explore the specific elements within campaigns to better isolate the effects of message content, delivery, intensity, focus and visual components, which may otherwise be conflated when assessed collectively, particularly among publicly accessible campaigns where confounds may not be sufficiently controlled. Specifically, research is needed to determine what delivery modes for messages could be used to improve the reach and sustainability of campaign promotions, with few studies examining the effect of social media despite its ability to increase engagement and communication with audiences (Neiger *et al*., 2012). The anonymity and ubiquity of social media potentially make it a feasible platform for disseminating targeted and repeat messaging over time to vulnerable populations (Torok *et al*., 2017), and more research is needed into its effectiveness on male help-seeking.

Males are not a homogenous group, and understandably, different male populations will interpret and respond differently to campaigns. Research is needed to determine whether optimal campaign approaches vary for particular male groups. While several included studies targeted younger males, only one appeared to target males aged 40–70 years (Wallhed Finn *et al*., 2023). Thus, it remains to be explored whether age-specific communication preferences influence the effectiveness of campaigns for older and younger adults, and additional research is warranted. Relatedly, despite the influence of masculine norms on male help-seeking (Levant *et al*., 2009), only one included study (Rochlen *et al*., 2006) examined whether adherence to masculine norms moderates the effectiveness of particular messages on male help-seeking outcomes. Thus, the influence of masculine norms in messaging represents an area for further research with important implications for enhancing male engagement in health-promoting behaviors.

## CONCLUSIONS

The current review indicates that media-based campaigns as a health promotion avenue can positively impact male help-seeking when addressing mental health or depression. However, the results should be interpreted with caution due to limitations in isolating the effects of campaign messaging from delivery and issues with heterogeneity of campaign approaches. Emerging evidence was found for using male or mixed-gender imagery, brochure-based campaigns and certain gain-framed messages such as those combined with male-sensitive language. There is an opportunity to develop future health promotion campaigns that optimize messaging and delivery tailored to such preferences to improve male help-seeking. Additional targeted studies evaluating the effectiveness of various components within media-based campaigns would assist with enhancing the evidence-based on interventions to improve male help-seeking for mental health.

## Supplementary Material

daae104_suppl_Supplementary_Material

## Data Availability

Data available within the article or its supplementary materials

## Appendix A

Example search string: PubMed

(Campaign*[tiab] OR ‘Health promotion*’[tiab] OR ‘Health promotion*’[Mesh] OR ‘Suicide prevention’[tiab] OR ‘Suicide prevention’[Mesh] OR ‘Health education’[tiab] OR ‘Health education’[Mesh] OR ‘Marketing’[tiab] OR ‘Marketing’[Mesh] OR ‘Public health messag*’[tiab] OR ‘Public health ad*’[tiab] OR ‘Promotion message’[tiab:~5] OR ‘Promotion messages’[tiab:~5] OR ‘Promotional messages’[tiab:~5] OR ‘Promotion messaging’[tiab:~5] OR Advertis*[tiab] OR Advertising[Mesh] OR ‘Public service announcement*’[tiab] OR Advocacy[tiab] OR ‘Communication strategy’[tiab:~5] OR ‘Communication strategies’[tiab:~5] OR ‘Communications strategy’[tiab:~5] OR ‘Communications strategies’[tiab:~5] OR ‘Health communications’[tiab:~5] OR ‘Health communication’[tiab:~5] OR ‘Public Health intervention’[tiab:~5] OR ‘Public Health interventions’[tiab:~5])

AND

(Media[tiab] OR Television[tiab] OR Television[Mesh] OR Radio[tiab] OR Radio[Mesh] OR Brochure*[tiab] OR Documentar*[tiab] OR Website*[tiab] OR Internet[tiab] OR Internet[Mesh] OR ‘Digital intervention’[tiab:~5] OR ‘Digital interventions’[tiab:~5] OR Announcement*[tiab] OR Film*[tiab] OR Movie*[tiab] OR ‘Motion pictures’[Mesh] OR ‘Motion picture*’[tiab] OR TV[tiab] OR Pamphlet*[tiab] OR ‘Video game*’[tiab] OR ‘Video games’[Mesh] OR Multimedia[tiab] OR News*[tiab] OR ‘Social media’[tiab] OR ‘Social media’[Mesh] OR Facebook[tiab] OR Twitter[tiab] OR Instagram[tiab] OR Weibo[tiab] OR Youtube[tiab] OR Telecommunication*[tiab] OR Telecommunications[Mesh] OR Music[tiab] OR Music[Mesh] OR Song*[tiab] OR ‘Mass media’[tiab] OR ‘Mass media’[Mesh] OR ‘Communication media’[tiab:~5] OR ‘Communications media’[tiab:~5] OR ‘Communications media’[Mesh] OR ‘Mass communication*’[tiab] OR Publication*[tiab] OR Publications[Mesh] OR Billboard*[tiab] OR poster*[tiab] OR flyer*[tiab])

AND

(‘Mental health’[tiab] OR ‘Mental health’[Mesh] OR ‘Mental ill*’[tiab] OR Psychiatr*[tiab] OR Distress*[tiab] OR ‘Psychological distress’[Mesh] OR ‘Mental disorder*’[tiab] OR ‘Mental disorders’[Mesh] OR ‘Mood disorder*’[tiab] OR ‘Mood disorders’[Mesh] OR ‘Emotional problem*’[tiab] OR ‘Alcohol use’[tiab] OR Depress*[tiab] OR Depression[Mesh] OR Anxiet*[tiab] OR Anxiety[Mesh] OR Suicid*[tiab] OR Suicide[Mesh] OR Psychosis[tiab] OR ‘Post-traumatic stress’[tiab] OR ‘Posttraumatic stress’[tiab] OR PTSD[tiab] OR ‘Substance use’[tiab] OR Bipolar[tiab] OR Bereavement[Mesh] OR Bereavement[tiab] OR Grie*[tiab] OR Grief[Mesh] OR Schizophrenia[tiab] OR Addiction[tiab] OR ‘Behavior, Addictive’[Mesh] OR ‘Mental disease’[tiab] OR ‘self harm*’[tiab] OR ‘self-harm*’[tiab] OR ‘Self-Injurious Behavior’[Mesh] OR ‘pathological gambling’[tiab] OR Gambling[Mesh])

AND

(‘Help-seeking’[tiab] OR ‘Help seeking’[tiab] OR ‘Seek help’[tiab:~5] OR ‘Seeking help’[tiab] OR ‘Service utilization’[tiab:~5] OR ‘Service utilisation’[tiab:~5] OR ‘Mental Health utilization’[tiab:~5] OR ‘Mental Health utilisation’[tiab:~5] OR ‘Service use’[tiab:~5] OR ‘Service usage’[tiab:~5] OR ‘Mental health use’[tiab:~5] OR ‘Mental health usage’[tiab:~5] OR ‘Mental Health Visit’[tiab:~5] OR ‘Mental health Visits’[tiab:~5] OR Care-seeking[tiab] OR ‘Care seeking’[tiab] OR ‘Treatment seeking’[tiab] OR ‘Treatment-seeking’[tiab] OR ‘Treatment pathway’[tiab] OR ‘Seek treatment’[tiab:~5] OR ‘Help seek*’[tiab] OR ‘Health acceptance’[tiab:~5] OR ‘Patient Acceptance of Health Care’[Mesh] OR ‘Help seeking behavior’[tiab] OR ‘Help-seeking behavior’[tiab] OR ‘Help-seeking behavior’[Mesh] OR ‘Help seeking behaviour’[tiab] OR ‘Help-seeking behaviour’[tiab] OR ‘Patient attitude*’[tiab] OR ‘Attitude to Health’[Mesh] OR ‘Health care utilization’[tiab:~5] OR ‘Health care utilisation’[tiab:~5] OR ‘Attitude mental health’[tiab:~5] OR ‘Attitudes mental health’[tiab:~5] OR ‘treatment barrier*’[tiab])
