# Correction to: The impact of media-based mental health campaigns on male help-seeking: a systematic review

**DOI:** 10.1093/heapro/daae151

**Published:** 2024-10-29

This is a correction to: Grant Duthie, Nicola Reavley, Judith Wright, Amy Morgan, The impact of media-based mental health campaigns on male help-seeking: a systematic review, *Health Promotion International*, Volume 39, Issue 4, August 2024, daae104, https://doi.org/10.1093/heapro/daae104

An error during production of this paper meant that Table 1, titled “The EPHPP quality assessment tool for quantitative studies” was omitted on first publication. This has been corrected, and the table published initially as Table 1 (“Campaign features of studies included in the review”) has been correctly identified as Table 3.

The publisher apologizes to the authors and to readers for the inconvenience caused.

**Table 1:** The EPHPP quality assessment tool for quantitative studies

Study	1. Selection bias	2. Design	3. Confounders	4. Blinding	5. Data collection methods	6. Withdrawals and dropouts	Global rating
Booth *et al*., 2018	S	M	S	S	S	S	S
Boucher and Campbell, 2014	W	S	W	W	S	S	L
Braun *et al*., 2023	W	S	S	S	S	S	M
Burns *et al*., 2010	W	M	W	W	S	W	L
Cheng *et al*., 2016	S	M	S	S	S	N/A	S
Daigle *et al*., 2006	S	W	W	W	M	N/A	L
Doss, 2000	W	S	W	M	S	N/A	L
Frey *et al*., 2023	W	S	S	M	S	M	M
Gilgoff *et al*., 2023	W	S	S	M	S	M	M
Hammer and Vogel, 2010	W	S	S	M	S	S	M
Hswen *et al*., 2022	W	W	M	W	M	W	L
King *et al*., 2018b	W	S	S	S	S	S	M
Maulik *et al*., 2019	M	M	M	W	S	S	M
Oliver *et al*., 2008	S	M	W	S	S	N/A	M
Rochlen *et al*., 2006	W	M	S	M	S	N/A	M
Schlichthorst *et al*., 2018	W	W	S	S	W	N/A	L
Shandley *et al*., 2010	W	M	W	W	S	W	L
Stas *et al*., 2023	W	M	M	W	S	W	L
Søgaard and Fønnebø, 1995	S	M	M	W	W	M	L
Till *et al*., 2013	S	M	W	S	S	N/A	M
Wallhed Finn *et al*., 2023	S	M	W	S	S	N/A	M

Component Ratings: 1A. Are the individuals selected to participate in the study likely to be representative of the target population? 1B. What percentage of selected individuals agreed to participate? 2A. Study design; 2B. Was the study described as randomised? 2C. Was the method of randomisation described? 2D. Was the randomisation process appropriate? 3A. Were there important differences between groups prior to the intervention? 3B. What percentage of relevant confounders were controlled? 4A. Were the outcome assessors aware of the intervention status of participants? 4B Were the participants aware of the research question? 5A. Were data collection tools shown to be valid? 5B. Were data collection tools shown to be reliable? 6A. Were withdrawals and drop-outs reported in terms of numbers/reasons? 6B. Indicate the percentage of participants completing the study. Global Rating Strong (S) = no weak (W) ratings; Moderate (M) = one weak rating; Low (L) = two or more weak ratings; N/A = not applicable.

