# Supplementary material for: The impact of media-based mental health campaigns on male help-seeking: a systematic review
Source: Health Promot Int. 2024 Sep 3;39(4):daae104. doi: 10.1093/heapro/daae104 (PMC11369358; doi:10.1093/heapro/daae104)
Supplement: daae104_suppl_Supplementary_Material [file daae104_suppl_supplementary_material.doc]

**Supplementary Table 1**

*Results of Studies Included in the Review*

| **Author (Date)** | **Study type** | **Outcome measures** | **Measurement points** | **Attitudes and beliefs, Intentions, or Behaviours** |
| --- | --- | --- | --- | --- |
| Booth et al. (2018) | ITSA | - The rate of outpatient mental health service visits to: - (1) Primary health care or - (2) Psychiatric services on a monthly basis. | Measured at preintervention, April 2008; peri-intervention, April 2011; and postintervention, April 2014. | **Behaviours**   - Primary care - Among primary care health care settings, adolescent males experienced a monthly visit increase from 9.7/1000 to 9.8/1000 (slope change of 0.05 following campaign, p < .001), while new visits saw a slope change of 0.01, p = .03. - No statistically significant change in visit rate magnitudes immediately after the campaign (p = .38). Young adult males experienced an increase in monthly visit rates to primary health care settings from 16.6/1000 to 20.3/1000 but there was a statistically significant immediate drop in young adult male visit rate magnitudes following the campaign (-2.9/1000, p < .001; slope change of 0.02, p = .28). - New visits saw a slope change of 0.01, p = .03 but a visit rate decrease following the campaign (-0.41, p = .003). - Outpatient psychiatric settings - Among outpatient psychiatric settings, adolescent males experienced a monthly visit increase from 2.5/1000 to 3.4/1000 (no change in rate, p = .88, slope change of 0.01, p < .001), while new visits increased from 0.74/1000 to 0.99/1000 (change in rate p = .65, slope change of 0.005, p = .02). - For young adult males an increase in psychiatric outpatient monthly visits from 1.95/1000 to 2.5/1000, but statistically significant immediate drop in psychiatric service visits (change = -0.32, p = .002), slop change of -0.01, p = .003). - New visits increased from 0.34/1000 to 0.4/1000 (p = .71, slope p = .23). |
| Boucher & Campbell (2014) | RCT | - Treatment willingness was assessed with three questions, on a 7-point Likert scale, asking participants to indicate their willingness to: - Seek any depression treatment - Take antidepressant medication and - Attend therapy/counselling. | Measured at post-test only. | **Intentions**   - Non-sig. interaction in gender by message (p NR). Non-sig. difference between groups in participant willingness to seek any depression treatment, take antidepressant medication, or go to therapy/counselling (p NR). |
| Braun et al. (2023) | RCT | - General Help-seeking Questionnaire including subscales for private and professional help-seeking intentions. - Higher scores indicate greater help-seeking intentions. | Measured at pretest, posttest, 4-weeks follow-up. | **Intentions**   - Help-seeking intentions - There was a non-sig. difference between groups for males (M diff = 0.00 (95% CI = -0.18 to 0.19), p = .96. Both groups showed a non-sig. increase over time. - Help-seeking private - There was a non-sig. difference between groups for males M diff = -0.22; 95% CI -0.48 to 0.04, p = .09. There was a sig. increase in the control group (M change = 0.28 (95% CI 0.09 to 0.48) and a non-sig. increase in the intervention group (M change = 0.16 (95% CI -0.05 to 0.38). |
| Burns et al. (2010) | Single-group quasi-experimental study | - As reported in Shandley et al. (2010), help-seeking intentions were assessed by asking participants to rate how likely it is they would seek help from various sources if they were feeling sad, down, or miserable for more than 2 weeks. - Direction of scale NR. | Measured at pretest, posttest, 2-month follow-up. | **Intentions**   - Additional publication of the results reported in Shandley et al. (2010): - Non-sig. WG change in help-seeking outcomes for males. Sig. WG change in help-seeking outcomes for females. |
| Cheng et al. (2016) | ITSA | - Change in monthly rate of psychiatric emergency department visits post-campaign including all visits with mental health and addiction main diagnostic codes in adults. | Measured at pretest and posttest. | **Behaviours**   - Among hospitals in Toronto downtown area in close proximity to CAMH: - Centre for Addiction and Mental Health increase of 7.6 PED visits/month post-campaign (p < .0001). At pre there were 1,832 males, at post there were 2,307 males. - University Health Network increase of 5.8 PED visits/month post-campaign (p < .0001). At pre there were 1,692 males, at post there were 2,072 males. - St. Michael's Hospital increase of 4.2 PED visits/month post-campaign (p < .0001). At pre there were 1,860 males, at post there were 2,185 males. - Mount Sinai Hospital increase of 3.2 PED visits/month post-campaign (p < .0001). At pre there were 1,010 males, at post there were 1,225 males. |
| Daigle et al. (2006) | Cross sectional study | - Help-seeking propensity (attitudes) were measured using six questions. - Help-seeking intentions was measured using two items. | Measured only at 1 week posttest. | **Attitudes**   - Non-sig. difference between those who had been exposed (M = 7.23, SD = 1.58) and not exposed (M = 7.06, SD = 1.61) to the campaign on help-seeking attitudes (p = .20).   **Intentions**   - Non-sig. difference between those who had been exposed and not exposed in intention of seeking help if they ever became suicidal (67% vs 63.7%, p NR). - Among men exposed to the campaign who had experienced suicidal thoughts (7% of the subgroup), only 54.5% had an intention to seek help, which was significantly lower compared to men with no suicidal thoughts (70.6%; p NR). |
| Doss (2000) | RCT | - Amenability to trying psychotherapy was measured using the Amenability to Trying Psychological Services (ATPS) Questionnaire. - Higher scores indicate a greater willingness to use the services for various problems. | Measured at post intervention only. | **Intentions**   - Sig. effect of brochure condition for ATPS F(2, 157) = 10.99 (p < .01), where gender sensitive brochure group M = 112.27 were more amenable to seeking psychological help than those from the non-gender sensitive brochure group M = 100.02 (p < .05, Cohen's d = 0.44) and C group = 85.59 (p < .05, Cohen's d = 0.94). - Participants given the gender-sensitive brochure indicated that they were more amenable to seeking psychological help than participants given either the non-gender-sensitive brochure or no brochure. - Participants who received the non-gender-sensitive brochure also were more amenable to seeking psychological help than no brochure participants (Cohen's d = 0.47). |
| Frey et al. (2023) | RCT | - Help-seeking intentions was assessed with the General Help-seeking behaviour for suicidality scale. - Higher scores represent greater likelihood of reaching out to family and friends. - Help-seeking attitudes was assessed with the Attitudes toward seeking professional psychological help scale. - Higher scores represent more positive attitudes. - Help-seeking behaviour was assessed with a single item asking participants if they had begun seeing a counsellor. | Measured at 2-weeks follow-up, 12-weeks follow-up. | **Attitudes**   - Non-sig. difference between conditions (p NR). - Both groups showed an improvement in their attitudes towards seeking help: Man Therapy M diff = 3.77, SD = 6.92; p = .004; 95% CI 2.70 to 4.84; Control group data NR.   **Intentions**   - Non-sig. difference between conditions (p NR). - Both conditions showed improvements in their intentions to reach out to people for help for suicidality: Man Therapy M diff = 2.29, SD = 9.97; p < .001; 95% CI 0.75 to 3.83); Control group data NR.   **Behaviours**   - Man Therapy participants reported increase in their help-seeking behaviours from 2-weeks (9%) to 12-weeks follow-up (17%), while those in the control reported increased help-seeking behaviour from 2-weeks (9%) to 12-weeks follow-up (19%; p's NR). - Non-sig. difference between conditions (p NR). |
| Gilgoff et al. (2023) | RCT | - To assess help-seeking behaviours participants selected from various checklist items all the different types of 8 professional and 5 non-professional help they have sought. - Converted to a dichotomous variable (sought any help vs not). | Follow-up measures conducted at 2 weeks post and 12 weeks after that. | **Behaviours**   - Secondary data analysis of Frey et al. (2023) - Binary logistic regressions of Man Therapy vs. control on professional help-seeking, where in the unadj. model was non-sig. OR = 1.43, 95% CI 0.94 to 2.18, p = .096, but in the adj. model (sexual orientation, education level, and marital status) was sig. OR = 1.55, 95% CI 1.00 to 2.40, p = .049. - The unadj. model for Man Therapy vs. control on nonprofessional help-seeking was non-sig. OR = 1.18, 95% CI 1.01 to 4.22, p = .497, and the adj. model (sexual orientation, education level, and marital status) was also non-sig. OR = 1.11, 95% CI 0.69 to 1.80, p = .658. |
| Hammer & Vogel (2010) | RCT | - Attitudes towards seeking psychological help was measured with the Attitudes Toward Seeking Professional Psychological Help Scale (ATSPPHS), short-form version. - Higher scores indicate more positive attitudes towards help-seeking. - Self-stigma towards seeking help was measured with the Self-Stigma of Seeking Help (SSOSH) scale. - Higher scores indicate greater self-stigma. | Measured at pretest and posttest. | **Attitudes and beliefs**   - Sig. interaction of time and brochure condition for attitudes F(2, 1308) = 3.5 (p = .03, partial eta 2 = 0.01), and self-stigma F(2, 1272) = 5.9 (p = .003, partial eta 2 = 0.01). - For attitudes, the male-sensitive brochure produced sig. greater improvements (M change = 0.12) than RMRD brochure (M change = 0.07) F(1, 871) = 6.7, (p = .01, partial eta 2 = .01), non-sig. difference with the gender-neutral brochure (M change = 0.08) (p = .086). - For self-stigma (beliefs), the male-sensitive brochure produced sig. greater improvements (M change = -0.09) than RMRD brochure (M change = 0) F(1, 837) = 12.2, (p < .001, partial eta 2 = .01), and gender-neutral brochure (M change = -0.01) F(1, 842) = 4.1, (p = .043, partial eta 2 = .01). |
| Hswen et al. (2022) | Cross-sectional survey | - A single question that asked participants about their intent of following up with a provider for treatment of depression after being provided with advice to seek help for symptoms of depression for those who scored greater than 16 on the Centre for Epidemiological Studies Depression (CES-D) scale, "In the coming week, do you plan to implement the consultation advice received?" | Measured at post-intervention. | **Intentions**   - Among those with a positive CES-D score, non-sig. difference in those willing to follow up with the consultation advise in the non-personalised group 20.4% compared to the personalised group 26% (p = .45). - This non-sig. difference was also shown in a probit regression model (p = .699), and one-tailed (p = .349). - In a probit regression model, there was a non-sig. difference in gender (male reference) on willingness to follow-up with the consultation advise (p = .603). |
| King et al. (2018a) | Qualitative feedback via online survey | - A survey questionnaire consisting of closed and open-ended questions was used to measure changes in attitudes and behaviours. | Measured at 4-weeks follow-up. | **Attitudes**   - After viewing the documentary, 70.0% of men (105) indicated that they had an increased confidence to reach out to someone. - "My attitude towards getting help definitely changed. I recognised that this is a health issue I have to change  and it’s easy to get help."   **Behaviours**   - After viewing the documentary, 41.3% of men (62) indicated that they had opened up to someone, 60.7% of men (91) indicated they had offered a friend time to talk, 38.7% of men (58) indicated they had encouraged a friend to seek help, and 10.7% of men (16) had seen a health professional for advice, 14% of men (24) indicated they had made change in reaching out to others. - "It made me reach out to my brother who is going through a difficult relationship breakdown and make sure he knows I’m here for him." - "I have noticed that when talking to other males I now pay more attention to what they are saying and how they are saying it, and have asked if they are ok more often." |
| King et al. (2018b) | RCT | - To measure help-seeking intentions the General Help Seeking Questionnaire (GHSQ-Self) was used. - Higher scores indicate higher intentions to seek help. - To measure intentions of recommending a friend seek help, the General Help Seeking Questionnaire-Modified (GHSQ-male friend, GHSQ-female friend) was used. - Higher scores indicate higher intentions to seek help. | Measured at baseline and 4-weeks follow-up. | **Intentions**   - There was a sig. interaction effect where the GHSQ-self score difference over time was higher in the intervention (M diff = 2.89) compared to the control group (M diff = 1.07), coef. = 2.06 (95% CI 0.48 to 3.63), p = .011, standardised mean difference=0.13. - There was also a sig. interaction effect favouring the intervention (M diff = 2.60) over the control group (M diff = 0.10) for the GHSQ-male friend coef. = 2.66 (95% CI 0.88 to 4.44), p = .004, standardised mean difference=0.15; and for the intervention (M diff = 1.71) over control group (M diff = -0.22) for the GHSQ-female friend coef. = 2.05 (95% CI 0.28 to 3.82), p = .023, standardised mean difference = 0.12. |
| Maulik et al. (2019) | Longitudinal study | - Stigma associated with seeking help for mental illness was measured with the Barriers to Access to Care Evaluation: Treatment Stigma subscale (BACE-TS version 3). - Higher scores indicate higher stigma. | Measured at pre-campaign, post-campaign, follow-up (24 months after the pre-campaign measurement). | **Attitudes**   - In a separate analysis of males who were interviewed at both visits (time 1 and 3), the difference over time was -0.3, which was sig. (p < .001). - Among males, scores at pre-campaign M = 0.37 (SD = 0.48), while at 24-months follow-up were M = 0.02 (SD = 0.11). |
| Oliver et al. (2008) | ITSA | - Suicide-related calls to an emergency mental health service. | Measured pre-campaign, during campaign, and between phases of the campaign. | **Behaviour**   - Non-sig. difference in proportion of calls made by gender during campaign months versus non-campaign months (p NR). - During the 15-month baseline period, there was an average of 23.1 ± 3.1 suicide calls per 100,000 residents per month. - During phase 1 of the campaign compared to baseline, average monthly rate increased by 29% to 29.9 ± 1.4 calls per 100,000 residents, which was a statistically sig. increase, p < .001 (95% CI difference between means, 4.5 to 9.1). - After phase 1, there was a sig. decrease in calls (p = .04), to 26.8 ± 2.0 (95% CI 0.1 to 6.2). During phase 2 compared to the hiatus period, there was a non-sig. increase in calls by 15% to 30.8 ± 3.4 (p = .07, 95% CI -0.4 to 8.4). - Sig. increase in mean monthly call rates during each month of the campaign compared to the corresponding month of the previous year for phase 1 (p = .01, M diff = 3.7, 95% CI 1.5 to 6.0) and phase 2 (p = .01, M diff = 9.5, 95% CI 4.2 to 14.7). |
| Rochlen et al. (2006) | RCT | - Help-seeking attitudes was assessed with the Attitudes Toward Seeking Professional Psychological Help Scale (ATSPPHS) short form. - Higher scores indicate more positive attitudes towards help seeking. | Measured at pre-intervention and post-intervention. | **Attitudes**   - Sig. difference over time for the gender-specific, average post-test score (1.6), t(68) = 4.3, p < .01, d = 0.35; non-gender specific, average post-test score (1.5), t(68) = 5.3, p < .01, d = 0.42; and depression brochures, average post-test score (1.5), t(65) = 2.7, p < .02, d = 0.17. - Non-sig. interaction between brochure and time, F = 1.7, p = .18, partial eta 2 = .02. Gender-role conflict was not a significant moderator of effects (p > .16). |
| Schlichthorst et al. (2018) | Repeat cross-sectional survey | - To assess help-seeking intentions, a single item asked participants their likelihood of asking help from someone they know or a healthcare service if they had a personal or emotional problem. | Measured at pre-screening and post-screening of the Man Up documentary. | **Intentions**   - Non-sig. difference between the pre-screening group and the post-screening who had not viewed Man Up coeff. (unadj.) = -0.31 (95% CI -0.62 to 0.00), p = .052; coeff. (adj. for age, country of birth, main language spoken at home, Aboriginal or Torres Strait Islander origin, sexual orientation, marital status, and experience of suicide of a male friend, colleague or family member) = -0.22 (95% CI -0.57 to 0.12), p = .209. - Non-sig. difference between the pre-screening group and the post-screening for those who had viewed Man Up coeff. (unadj.) = 0.02 (95% CI -0.21 to 0.25), p = .853; coeff. (adj. for age, country of birth, main language spoken at home, Aboriginal or Torres Strait Islander origin, sexual orientation, marital status, and experience of suicide of a male friend, colleague or family member) = 0.09 (95% CI -0.17 to 0.35), p = .485. |
| Shandley et al. (2010) | Single-group quasi-experimental study | - Help-seeking intentions were assessed by asking participants to rate how likely it is they would seek help from various sources if they were feeling sad, down, or miserable for more than 2 weeks. - Direction of scale NR. | Measured at pre, post, and 2-month follow-up. | **Intentions**   - There was a significant improvement over time in help-seeking intentions, F(1,264) = 18.04, p < .001 (partial eta 2 = 0.06, beta-1 = 0.99), and a significant interaction (time x gender) at post-intervention F(1,264) = 6.12, p = .01 (partial eta 2 = 0.02, beta-1 = 0.70), where the increase in willingness to seek help was greater among females than males. |
| Stas et al. (2023) | Cohort study | - To measure help-seeking intentions for personal or emotional problems, as well as when experiencing suicidal thoughts, the General Help-seeking Questionnaire was administered. - Direction of scale NR. | Measured at pretest and post-test. | **Intentions**   - Non-sig. difference in help-seeking intentions overall from pre-test (M = 64.33, SE = 1.01) to post-test (M = 65.81, SE = 1.13), 95% CI -0.11 to 3.08, p = .068, d = 0.11. - However, there was a small increase from pre-test (M = 30.67, SE = 0.60) to post-test (M = 31.88, SE = 0.68) in help-seeking intentions when experiencing suicidal thoughts (95% CI 0.17 to 2.25, p = .023, d = 0.15). |
| Søgaard & Fønnebø (1995) | Cohort study | - Attitudes towards help-seeking was measured using a single item asking about which sources of help a person would recommend to others. - To measure help-seeking intentions, a single question was asked about whether participants would hesitate to seek help if their GP recommends them to see a psychiatrist or psychologist. | Measured at 5 months pre-campaign, 1-months post-campaign. | **Attitudes**   - After the campaign, more males reported they would recommend professional help from a GP for someone else (18.9% vs 31.3%, p < .001). - There was no sig improvement in recommending a psychiatrist or psychologist (10.3% vs 11.0%, p NR) or vicar (1.1% vs 3.6%, p NR).   **Intentions**   - After the campaign, there was a sig. decrease in for both sexes combined in those who would hesitate to see a psychiatrist/psychologist (p < .01). - However, there was a non-sig. change for males (pre-campaign 20.1% to post-campaign 14.7%) or females (pre-campaign 18.6% to post-campaign 13.4%) when analysed separately (p's NR). |
| Till et al. (2013) | Cohort pre- and postintervention comparison | - Phone calls to a telephone crisis hotline. | Measured at 3 months pre-campaign and 3 months post-campaign. | **Behaviours**   - Sig. increase in overall phone calls in the study region compared to control region (chi 2 = 12.96, df = 1, p < .001). Increase in calls in the study region from 4,439 in the control period to 4,649 (+4.7%) and in the control region phone calls decreased from 5,152 to 4,861 (-5.6%) during the same period. - However, the increase in the study region was non-sig. different to other increases in calls in 2009 (chi 2 = 0.42, df = 1, p = .51) and sig. lower than calls in 2010 (chi 2 = 5.47, df = 1, p < .05). - Non-sig. gender specific change in suicide-related calls of men (study region: pre = 7, post = 1, control region: pre = 10, post = 2, p = 1.00) and women (study region: pre = 13, post = 7, control region: pre = 6, post = 10, p = .17). |
| Wallhed Finn et al. (2023) | ITSA | - Changes in treatment seeking for alcohol use disorder (prescriptions filled or entries to treatment). | Measured at pre-campaign, campaign period, between or after campaign period. | **Behaviours**   - For men, entries to treatment (IRR = 0.92 (95% CI 0.86 to 0.98), p = .010) and prescriptions filled (IRR = 0.87 (95% CI 0.77 to 0.97), p = .016) was lower between/after campaigns compared to before. - Non-sig. difference for males when comparing before campaigns to during the campaign period for entries to treatment (p = .778) and prescriptions filled (p = .082). - When stratifying on primary target group of men aged 40-70, sig. difference when comparing between or after campaigns to before any campaign (Entries to treatment: IRR = 0.87 (95% CI 0.82 to 0.93), p= < .001; Prescriptions filled: IRR = 0.87 (95% CI 0.79 to 0.96), p = .004. - Sig. difference for this age group for prescriptions filled comparing the campaign period to before campaigns IRR = 0.89 (95% CI 0.80 to 0.99), p = .039, but non-sig. difference for entries to treatment (p = .202). |

Notes. RCT = Randomised Controlled Trial; ITSA = Interrupted Time Series Analysis; M = mean; Pre = pre-test measures, Post = post-test measures; Sig = significance; df = degrees of freedom; CI = confidence interval; Adj = adjusted; Unadj = unadjusted; Coef = coefficient; HS = help-seeking; Diff = difference; IRR = Internal rate of return; NR = not reported
